# Parenting style and the non-cognitive development of high school student: evidence from rural China

**DOI:** 10.3389/fpsyg.2024.1393445

**Published:** 2024-07-18

**Authors:** Sangui Wang, Lijuan Zheng

**Affiliations:** ^1^School of Agricultural Economics and Rural Development, Renmin University of China, Beijing, China; ^2^China Anti-poverty Research Institute, Renmin University of China, Beijing, China

**Keywords:** non-cognitive abilities, authoritative parenting, authoritarian parenting, high school student, rural China

## Abstract

**Introduction:**

Understanding the relationship between parenting style and the non-cognitive development of high school students is crucial, particularly in rural China. Non-cognitive abilities, including traits such as emotional regulation, resilience, and interpersonal skills, play a significant role in students’ overall development and future success. This study aims to investigate how different parenting styles impact non-cognitive abilities among high school students in rural China.

**Methods:**

This study surveyed 6,549 high school students and their primary caregivers in rural China. The students had an average age of 17.61 years, with 48% being male, and 62% of Han ethnicity. Primary caregivers self-reported their parenting styles, while the students’ non-cognitive abilities were assessed using the Big Five Inventory-Short (BFI-S). The relationship between parenting style and non-cognitive development was analyzed using two distinct methods: two dimensions (authoritative and authoritarian) and four categories of parenting styles.

**Results:**

The study revealed that an authoritative parenting style had a positive impact on the non-cognitive abilities of students. Conversely, a negative association was observed between the authoritarian parenting style and the students’ non-cognitive development. This association was more pronounced in the non-cognitive developmental scores of girls compared to boys. Additionally, parents from wealthier families or those with higher levels of education were more likely to adopt an authoritative parenting style rather than an authoritarian one.

**Discussion:**

The results of this study highlight the significant influence of parenting styles on the non-cognitive development of high school students in rural China. Authoritative parenting, characterized by warmth and structure, appears to foster better non-cognitive outcomes, while authoritarian parenting, marked by strictness and less warmth, is associated with poorer non-cognitive development. The gender differences observed suggest that girls may be more sensitive to variations in parenting style. Furthermore, the socioeconomic and educational background of parents plays a crucial role in determining the parenting style adopted. These findings underscore the importance of developing and implementing parenting training interventions in rural China, aimed at promoting authoritative parenting practices to enhance the non-cognitive development of students.

## 1 Introduction

The human capital literature emphasizes the crucial role of non-cognitive skills in shaping long-term economic outcomes. The development of non-cognitive abilities, encompassing traits like motivation, perseverance, interpersonal skills, self-esteem, and emotional regulation during childhood, establishes the groundwork for various life outcomes such as educational attainment, adult health conditions, labor market performance, and earnings (Heckman and Rubinstein, 2001; Cunha et al., 2010; Almlund et al., 2011; Kautz et al., 2014). Moreover, non-cognitive abilities have been recognized as more malleable than cognitive abilities. Specifically, cognitive skills undergo the greatest amount of change in early childhood and stabilize by adolescence. In contrast, non-cognitive skills continue to develop throughout childhood and into young adulthood, indicating a greater potential for improvement during later developmental stages (Brunello and Schlotter, 2011; Gutman and Schoon, 2013; Hoeschler et al., 2018). Given the importance and malleability of non-cognitive abilities, researchers have been motivated to investigate the determinants of school-aged children’s non-cognitive development, with a particular focus on the role of the family, which is acknowledged as a significant contributor to children’s skill formation (Becker and Tomes, 1986).

The impact of familial factors on the non-cognitive development of school-aged children exhibits a high level of complexity, with both school and family factors playing pivotal roles in shaping student non-cognitive abilities (Cunha and Heckman, 2007; Veiga et al., 2023). While numerous studies focus on investigating the influence of specific aspects of family characteristics, such as household income or socioeconomic status (Blau, 1999; Loken et al., 2012), parental education level (Leight and Liu, 2020), parental time and material investment (James-Burdumy, 2005; Bernal and Keane, 2011), and the child’s birth order (Hotz and Pantano, 2015), the impact of parenting style on children’s non-cognitive development has received comparatively less attention.

The concept of parenting style, as developed by Baumrind (1967, 1971), indicates how parents respond to their children’s needs or behaviors. Parenting styles are defined by two main dimensions: responsiveness and demandingness, which are theoretically orthogonal or unrelated (Lamborn et al., 1991; Fuentes et al., 2022). Responsiveness involves parental warmth, involvement, and support for the child’s individuality (Baumrind, 2013; Alcaide et al., 2023). Demandingness refers to the degree of strictness and the expectations parents have for their child to conform to society and family standards (Martinez-Escudero et al., 2020). Baumrind (1967) initially identified three primary parenting styles: authoritative, authoritarian, and permissive. Authoritative parenting combines high demands with high responsiveness and is associated with greater parental involvement, trust, and support (Durbin et al., 1993). Authoritarian parenting is characterized by high demands and strict control, but low responsiveness and communication (McClun and Merrell, 1998). Permissive parenting involves high parental warmth and a child-centered approach but lacks discipline (Smetana, 1995; Villarejo et al., 2024). Studies have shown that Baumrind’s classification can be insufficient and has limitations in both Western and Eastern contexts (Darling and Steinberg, 2017; Chen et al., 2024). McCoby (1983) extended the framework by categorizing the permissive parenting style into negligent and indulgent. Neglectful parenting is defined by a lack of expectations and attentiveness, when parents demonstrate minimal concern for their children’s viewpoints, pursuits, and feelings (Climent-Galarza et al., 2022; Palacios et al., 2022). Indulgent parenting is defined by a significant degree of attentiveness to the needs and wants of children, but a lack of emphasis on requiring and expecting adult behavior (García and Gracia, 2013).

The link between parenting style and students’ cognitive and academic achievements has been well-established (Spera, 2005; Brown and Iyengar, 2008; Dornbusch et al., 2016; Xia, 2020)^1^. While evidence supporting the relationship between parenting style and non-cognitive child development has emerged more recently, earlier studies primarily focused on aspects such as the child’s locus of control, risky behavior, patience, risk aversion, altruism, and social skills (Aunola and Nurmi, 2005; Alegre, 2011). For instance, Cobb-Clark et al. (2019) affirmed that respectful parenting correlated with an increased internal locus of control and a decreased inclination toward risky behavior. Fiorini and Keane (2014) demonstrated the significant impact of parenting style on non-cognitive abilities, encompassing behavioral problems, social skills, and emotional issues. In a recent study, Falk et al. (2021) explored the relationship between parenting style and a child’s patience, risk aversion, conduct, and altruism. Their findings highlighted that a parenting style characterized by warmth and child-centeredness positively influenced all these aspects. Several studies consistently indicate that a parenting style combining effective disciplinary practices with parental warmth leads to the highest child adjustment (Martinez-Escudero et al., 2023).

This study aims to expand and enhance existing research on the relationship between parenting style and the non-cognitive development of high school students. Previous studies have shown that non-cognitive skills may be broadly defined as personality traits or “patterns of thought, feelings, and behavior” (Borghans et al., 2008), encompassing a broad range of characteristics, such as personality traits, motivation, confidence, perseverance, and social and communication skills (Hoeschler et al., 2018). We utilize a comprehensive scale that assesses a broader spectrum of personality traits related to non-cognitive abilities. Heckman and Kautz (2012) contend that, despite the diverse nature of non-cognitive abilities, the Big Five^2^—widely investigated in psychology—can effectively serve as an assessment tool for these abilities. The Big Five personality traits encompass openness, conscientiousness, extraversion, agreeableness, and emotional stability. Therefore, our objective is to explore the potential correlation between parenting style and the non-cognitive development of high school students. We categorize parenting styles using both a two-dimensional and a four-dimensional framework. To achieve this, we classify students into four groups based on high and low levels of authoritative and authoritarian parenting styles. Our investigation centers on understanding how these four distinct parenting styles impact students’ non-cognitive development.

While the global evidence base connecting parenting style to non-cognitive development continues to grow, there remains a scarcity of evidence regarding parenting style and children’s non-cognitive abilities. Presently, only two studies have delved into this area. Kugler et al.’s (2022) research, to the best of our knowledge, is the first to explore the relationship between parenting style and children’s non-cognitive ability in developed countries, specifically Germany. Another study, conducted in Western Europe (Loudová and Lašek, 2015), also addresses this topic. Both studies had limited sample sizes^3^, and were not conducted in Asian countries, where parents often exhibit a higher degree of disciplinary behavior (Deng and Tong, 2020). Additionally, neither study used samples from rural areas. Moreover, research has also revealed that cultural context may influence the prevalence and outcomes of different parenting styles (Pinquart and Kauser, 2018; Chen et al., 2024). Western cultures value individuality and self-expression, resulting in different effects of authoritarian and permissive parenting styles (Reyes et al., 2023). For example, studies conducted mainly in European and South American countries identified benefits related to greater responsiveness but without demandingness (Garcia et al., 2019). Chinese culture, shaped by Confucianism, emphasizes respect for authority and academic achievement (Chao, 1994). Studies within Chinese American families have shown that authoritarian parenting is related to benefits, especially in academic achievement (Chao, 2000). Therefore, the primary contribution of our study is to augment the existing literature by gathering data on caregivers’ parenting styles and their potential impact on children’s non-cognitive abilities in rural China.

A second noteworthy contribution of this study is the expansion of the age range within the sample population. Previous studies have established correlations between parenting style and the non-cognitive development of young children. Specifically, authoritarian parenting has been associated with increased extraversion and openness, while authoritarian-inconsistent parenting has been linked to heightened extraversion, conscientiousness, agreeableness, and increased emotional stability (Kugler et al., 2022). Ashraf et al. (2018) also reported a causal relationship between parenting style and personality traits in primary school children. However, it is important to highlight that none of the existing research has explored the impact of parenting style on the Big Five personality traits of high school students. Although adolescents seek greater independence, parental style continues to play a crucial role in shaping their non-cognitive skills (Zhang and Wang, 2022). Moreover, parenting style evolves with the child’s age (Burnett et al., 2021) and may have different impacts on the non-cognitive abilities of children at different ages (Rosen et al., 2008). Parenting typically diminishes as the child reaches adulthood, at which point parents can no longer employ responsiveness and demandingness (Máñez et al., 2024).

The objective of this study is to investigate the relationship between parenting style and non-cognitive development outcomes among high school students, utilizing a substantial dataset and incorporating Big Five measures to assess non-cognitive development.

A third significant contribution is our examination of the differential impact of caregivers’ parenting styles on children’s non-cognitive development based on gender. Studies have indicated variations in the relationships between parenting style and child non-cognitive outcomes when considering gender differences (Crouter et al., 1995; Braza et al., 2015). Existing literature suggests a moderate role for a child’s gender in the dynamic interaction between parenting style and students’ non-cognitive development (Deater-Deckard et al., 2003; Barnett and Scaramella, 2013). One theory of child socialization posits that parents respond differently to boys and girls, adopting distinct parenting approaches for each gender. Additionally, the differential susceptibility theory suggests that different genders may react differently to the same parenting style (Keshavarz et al., 2012; Mandara et al., 2012). For example, Mandara et al. (2012) found that mothers exhibited greater warmth and support towards their daughters than their sons, with these parenting disparities contributing to more problematic behaviors in boys. Keshavarz et al. (2012), in a study involving 382 children and their parents in Malaysia, discovered that boys, especially those raised with authoritative fathers, exhibited better developmental outcomes compared to girls. This study also aims to investigate the heterogeneous effect of parenting styles on high school students’ non-cognitive development, considering gender differences.

The remainder of the paper is structured as follows. The sample selection, data collection, ethical review, and econometric framework are described in Section 2. The results are presented in Section 3, and Section 4 concludes.

## 2 Methods

### 2.1 Sample selection

The data for this study were gathered through a survey conducted among high school students and households in two counties within Haidong City, located in Qinghai Province in 2023. Qinghai, predominantly situated on the Tibetan Plateau in northwestern China, is renowned for its high altitude and diverse mountainous terrain. Although geographically expansive, Qinghai is one of China’s most sparsely populated provinces, with only 5.9 million residents, ranking second-fewest in population. Within this population, 49.47% belong to ethnic minority groups, and 58.8% reside in rural areas. In terms of GDP *per capita*, Qinghai ranks second to last among China’s provinces. For our study, we randomly selected two counties, Ledu and Minhe, from six within Haidong City. Both counties, situated in eastern Qinghai, were designated as national-level poverty-stricken areas by the China State Council in 2012 and successfully emerged from poverty in 2020.

Following the identification of the specific locations of the sample counties, the research team initially acquired the roster of all students attending high schools in Ledu and Minhe counties from the local Bureau of Education office. Ledu County comprises four high schools, consisting of three ordinary high schools and one vocational high school. Similarly, Minhe County is home to five high schools, including four ordinary high schools and one vocational high school.

Utilizing a comprehensive student list, our objective was to inclusively select all students and their families registered in the high schools. In June 2023, we identified and obtained consent from 6,560 students and households to participate in our study. Out of the 6,560 enrolled student-caregiver dyads, 11 were excluded from the analysis due to incomplete data regarding the parenting style of the caregiver; these caregivers either declined or were unable to complete the survey form. Consequently, the total number of student-caregiver dyads in our sample is 6,549.

### 2.2 Data collection

In anticipation of the data collection phase, we recruited a total of 20 enumerators in April 2023. Enumerators were selected from postgraduate students at universities in Beijing and Qinghai Province. All enumerators underwent a comprehensive three-day training program, emphasizing the principles and techniques of survey administration. This training was conducted by a team of fieldwork professionals, including both enumerators and team leaders.

Data collection took place over a two-week period in May 2023, involving nine data collection teams. Each team, comprising a trained enumerator and a team leader, administered questionaires to students and primary caregivers. Vocational high schools have more classes, so the two vocational schools each had a trained enumerator, a team leader, and an assistant. All data collection procedures were conducted in the computer labs of each high school, with student and caregiver questionnaires completed under the supervision of an enumerator. The process involved alternating between one class and another. Initially, we gathered student questionnaires from each high school, obtaining data on the non-cognitive development of students. Subsequently, the school notified the primary caregiver of each student by class, who then completed primary caregiver questionnaires at the school. The primary caregiver, identified as the individual responsible for the student’s daily care and nutrition, is typically a parent or grandparent. Information on parenting styles was collected from the primary caregivers’ questionnaires.

#### 2.2.1 Non-cognitive development of high school students

The non-cognitive abilities of students were assessed using the Big Five Inventory-Short (BFI-S), developed by Gerlitz and Schupp (2005). The Big Five model, widely accepted for describing personality (John et al., 2008), categorizes personality into five fundamental traits, represented by the acronym OCEAN: Openness refers to the tendency to be curious and pursue intellectual interests, reflecting an individual’s inclination toward exhibiting imaginative, creative, unconventional, emotionally perceptive, and aesthetically sensitive qualities. Conscientiousness is the tendency to be hardworking and organized, pertaining to an individual’s inclination toward being organized, possessing strong willpower, demonstrating persistence, exhibiting reliability, and adhering to laws and ethical principles. Extroversion is the tendency to be outgoing and sociable, signifying an individual’s inclination toward sociability, warmth, activity, assertiveness, cheerfulness, and the pursuit of stimulation. Agreeableness is the tendency to be unselfish and friendly, encompassing the interpersonal component characterized by altruistic tendencies, trustworthiness, modesty, and cooperativeness. Emotional stability is the tendency to have consistency in emotional reactions.

As recommended by Hahn et al. (2012), we employed a BFI-S consisting of 15 items, with three items allocated to each personality dimension (detailed items can be found in Supplementary Table 1A). Participants rated their agreement with each statement on a 5-point Likert scale ranging from 1 = “strongly disagree” to 5 = “strongly agree.” The original Spanish BFI-S form was translated into Mandarin Chinese by a native speaker and utilized in a comprehensive survey conducted in China, known as CFPS^4^ (Wu and Gu, 2020). Following Wu and Gu’s (2020) methodology, we adhered to the scoring procedure utilized by other researchers (Dehne and Schupp, 2007). Specifically, we evaluated the BFI-S dimensions using three items per dimension. Conscientiousness, extraversion, agreeableness, openness, and emotional stability are all positive indicators, meaning that high scores indicate high levels of non-cognitive abilities. In our sample, we assessed the internal consistency reliability of the BFI-S using Cronbach’s alpha coefficient, revealing acceptable internal consistency among the caregivers with a Cronbach’s alpha of 0.71. In the empirical analysis, the standardized score of five major dimensions was utilized.

#### 2.2.2 Parenting style of primary caregivers

To evaluate parenting style, we administered the Parenting Styles and Dimensions Questionnaire-Short Version (PSDQ-S) survey to the primary caregivers of all sample students. The PSDQ-S questionnaire, developed by Robinson et al. (2001), serves to measure the parenting style of caregivers. The PSDQ-Short Version comprises six subgroups: three for authoritative and three for authoritarian parenting styles. The three components of an authoritative parenting style include Connection (warmth and support), Regulation (reasoning and induction), and Autonomy Granting (democratic participation). For authoritarian parenting style, the three elements are Physical Coercion, Verbal Hostility, and Non-Reasoning or Punitiveness. Due to the observed low reliability of indulgent and neglectful parenting style constructs within the Chinese cultural context (Chan et al., 2009; Ren and Pope Edwards, 2015; Wang et al., 2022), and the low prevalence of these styles among parents in China (Wu et al., 2002; Li and Xie, 2017), the data we collected does not include items related to permissive and neglectful parenting styles.

The PSDQ-S questionnaire comprises 27 items, requiring participants to assess the extent of their engagement in parenting activities by rating statements on a 5-point Likert scale, ranging from “never” (1) to “always” (5). We scored the PSDQ-S version using the same method as employed in prior studies (Kern and Jonyniene, 2012; Fu et al., 2013). Specifically, we utilized the Baumrind (1967) typologies, encompassing 15 questions measuring authoritative parenting and 12 measuring authoritarian parenting. A higher score on each dimension indicates a higher frequency of parenting practices aligning with the corresponding parenting style. In our study, we assessed the internal consistency and reliability of the PSDQ-Short Version using Cronbach’s alpha coefficient. The results indicated alpha coefficients of 0.90 for the authoritative parenting style dimension, 0.89 for the authoritarian parenting style dimension, and 0.88 for the overall PSDQ scale.

#### 2.2.3 Student and household characteristics

Data on student and household characteristics were gathered through both a student-report questionnaire and a parent-report questionnaire. For student characteristics, we recorded their age in years, gender, boarding status, Hukou status (whether rural or not), and minority affiliation (Han or not). Additionally, we collected data on household characteristics, including family size, the number of siblings the student has, the relationship of the primary caregiver to the student (e.g., parents or grandparents), whether the household is categorized as poverty-stricken, paternal and maternal education levels, and household assets (e.g., whether the household had internet access or a flush toilet at home).

### 2.3 Statistical analysis

We conducted an analysis to examine the correlation between the parenting style of caregivers and high school students’ non-cognitive development outcomes. As detailed in Section 2.2, parenting style is classified into authoritative and authoritarian categories to estimate these associations.

To achieve this objective, we initially estimate the relationship between specific subscales of parenting style and non-cognitive development outcomes. The regression specification is computed using the ordinary least squares (OLS) regression method (see Equation 1).


(1) $$Y_{i,c}=\alpha_{0}+\alpha_{1}A_{i,c}+\eta X_{i,c}+\varepsilon_{i,c}$$

where the dependent variable, *Y_i,c_*, refers to students’ non-cognitive abilities: The standardized score of five specific dimensions of BFI-S. The variable A_i,c_ represents the authoritative or authoritarian parenting style score of student *i* in class c. *X_i,c_* refers to student and household characteristics. Standard errors in all regression specifications are adjusted for clustering at the class level.

Second, following previous studies (Liu and Lachman, 2019), we put both authoritative and authoritarian parenting styles in the model to obtain the following OLS regression specification (see Equation 2):


(2) $$Y_{i,c}=\alpha_{0}+\alpha_{1}A_{1,i,c}+\alpha_{2}A_{2,i,c}+\eta X_{i,c}+\varepsilon_{i,c}$$

A_1,i,c_ represents the authoritative parenting style score of student *i* in class c, and the A_2,i,c_ represents the authoritarian parenting style score of student *i* in class c.

We further investigate differences in the relationships of parenting styles between boys and girls. For this exploratory analysis, we run the following OLS regression specification (see Equation 3):


(3) $$\begin{array}{c} Y_{i,c}=\alpha_{0}+\lambda_{1}G_{i,c}+\alpha_{1}A_{1,i,c}+\beta_{1}\left(A_{1,i,c}G_{i,c}\right)+\alpha_{2}A_{2,i,c}\\ +\;\beta_{2}\left(A_{2,i,c}G_{i,c}\right)+\eta X_{i,c}+\varepsilon_{i,c} \end{array}$$

where G_i, c_ is a dummy indicator that takes the value of 1 if the student is male and the value of 0 if the student is female.

To further explore the correlations between various subgroups based on combinations of parenting styles—authoritative and authoritarian—with student non-cognitive development, we adopted a four-dimensional approach to classify parenting styles into more specific subgroups. To compare different combinations of “high” and “low” values for the two parenting styles, students were grouped based on their caregivers’ ratings on the authoritative and authoritarian subscales, as established in previous studies (Zhang and Qin, 2019). Group 1 (high authoritative, low authoritarian) comprised caregivers whose ratings on the authoritative subscale were higher than the median but ratings on the authoritarian subscale were lower than the median. Group 2 (low authoritative, high authoritarian) included caregivers with ratings on the authoritative subscale below the median and ratings on the authoritarian subscale above the median. Caregivers scoring higher than the median on both the authoritative and authoritarian subscales were classified as Group 3 (high authoritative, high authoritarian). Group 4 consisted of parents and caregivers scoring below the median on both the authoritative and authoritarian dimensions, serving as a comparison group since their parenting methods were less authoritative and less authoritarian than the norm. For a comprehensive robustness assessment of the analysis, students were additionally categorized into four distinct groups based on cutoff values derived from the mean scores of the authoritative and authoritarian subscale ratings. The regression specification is estimated using the ordinary least squares (OLS) regression method (see Equation 4):


(4) $$Y_{i,c}=\alpha_{0}+\alpha_{1}P_{2,i,c}+\alpha_{2}P_{2,i,c}+\alpha_{3}P_{3,i,c}+\eta X_{i,c}+\varepsilon_{i,c}$$

where P_1,i,c_ is a dummy indicator that takes the value of 1 if the primary caregiver takes high authoritative and low authoritarian parenting styles and the value of 0 if else; P_2,i,c_ is a dummy indicator that takes the value of 1 if the primary caregiver takes low authoritative and high authoritarian parenting styles and the value of 0 if else; and P_3,i,c_ is a dummy indicator that takes the value of 1 if the primary caregiver takes high authoritative and high authoritarian parenting styles and the value of 0 if else. Standard errors are also adjusted for clustering at the class level.

To investigate the potential associations between specific student and household characteristics and authoritative and authoritarian parenting styles, we employed the following model (see Equation 5):


(5) $$A_{i,c}=\beta_{0}+\beta_{1}X_{i,c}+\mu_{i,c}$$

where A_i,c_ represents the dependent variable (which is either authoritative or authoritarian parenting style score of the primary caregiver of student i). As in the model above, the variable X_i,c_ is a vector of covariates of student and household characteristics and μ_i,c_ is an error term.

## 3 Results

### 3.1 Student and household characteristics

Table 1 presents the socioeconomic and demographic characteristics of the sample students. The average age of the students was 17.61 years, ranging from 16 to 18. Approximately half of the students (48%) were male; 48% of the students resided in school, 75% had rural hukou, and 62% were Han minority. When examining household characteristics, the data revealed that the average family size of the sample students is 4.73, the average number of siblings per student is 1.12, and in 93% of sample students, parents were the primary caregivers. Additionally, 12% of sample students were from poverty-stricken households, and only 23% of fathers and 17% of mothers had completed upper secondary education or above. The average score for authoritative parenting style, as reported by 6,549 caregivers regarding the students, was 3.32, while the average score for authoritarian parenting style was 2.16.

**Table 1:** Summary statistics for students and households.

**Variables**	**Mean (SD)/Percentage**
**Student characteristics**	
(1) Age (in years)	17.61 (1.19)
(2) Gender	
Male	48%
Female	52%
(3) Student is boarding	
Yes	48%
No	52%
(4) Hukou is Rural	
Yes	75%
No	25%
(5) Minority is Han	
Yes	62%
No	38%
**Household characteristics**	
(6) Family size (numbers)	4.73 (1.32)
(7) Siblings (numbers)	1.12 (0.79)
(8) Primary caregiver is parents	
Yes	93%
No	7%
(9) Paternal education level (years)	
<12	77%
> = 12	23%
(10) Maternal education level (years)	
<12	83%
> = 12	17%
(11) Poverty-stricken household	
Yes	12%
No	88%
(12) Family asset index	0.04 (1.56)
**Parenting style**	
(13) Authoritative	3.32 (0.71)
(14) Authoritarian	2.16 (0.67)

The table shows the mean and the standard deviation for age (row 1), family size (row 5), the number of siblings of students (row 6), family asset index (row 11), Authoritative score (row12), and Authoritarian score (row13), while shows the percent for other indictors. The construction of the family asset index involved the utilization of polychoric principal component analysis, which was based on a set of variables including tap water, toilet facilities, water heater, washing machine, television, computer, internet access, refrigerator, microwave oven, extractor, air conditioner, motor or electric bicycle, and car.

### 3.2 Non-cognitive outcomes of high school student

Table 2 presents the high school students’ non-cognitive developmental subscale scores in the full sample and sub-samples. The dimension scores for conscientiousness, extroversion, agreeableness, openness, and emotional stability are 3.32 (0.63), 3.27 (0.68), 3.65 (0.61), 3.54 (0.68), and 2.85 (0.70), respectively.

**Table 2:** Non-cognitive score of sampling high school student.

	**Full sample**	**Boys**	**Girls**	***p*-value (boys vs. girls)**
	**Mean (SD)**	**Mean (SD)**	**Mean (SD)**	
**Non-cognitive abilities**				
Conscientiousness	3.32	3.37	3.25	0.000
	(0.63)	(0.67)	(0.60)	
Extroversion	3.27	3.29	3.25	0.039
	(0.68)	(0.68)	(0.68)	
Agreeableness	3.65	3.66	3.64	0.269
	(0.61)	(0.60)	(0.60)	
Openness	3.54	3.60	3.48	0.480
	(0.68)	(0.69)	(0.67)	
Emotional stability^1^	2.85	2.97	2.73	0.000
	(0.70)	(0.69)	(0.70)	
Observations	6,549	3,117	3,432	

^1^In order to keep consistency with the scores of other dimension of non-cognitive ability, the emotional stability dimension is also adjusted to a positive indicator, meaning that the higher the score, the more stable the student’s emotions are.

A comparison of the sub-samples of boys and girls reveals distinctions in non-cognitive outcomes. Specifically, boys, with a conscientiousness score of 3.37, an extroversion score of 3.29, an agreeableness score of 3.66, an openness score of 3.60, and an emotional stability score of 2.97, outperform girls in these subscales. Additionally, boys have substantially higher scores for extraversion, conscientiousness, and emotional stability.

### 3.3 Parenting style and non-cognitive outcomes

#### 3.3.1 Two dimensions of parenting style and students’ non-cognitive outcomes

The correlations between authoritative or authoritarian parenting style and student non-cognitive developmental outcomes as measured by the BFI-S are presented in Table 3. Notably, when controlling for student- and household-specific variables, authoritative measurement scores were significantly and positively associated with all sub-indexes at the 1% significance level, except for emotional stability. A one-point increase in authoritative measurement scores was linked to an increase in conscientiousness, extroversion, agreeableness, and openness by 0.108 SD, 0.069 SD, 0.101 SD, and 0.123 SD, respectively. In contrast, authoritarian measurement scores were significantly and negatively correlated with student non-cognitive specific sub-indexes (conscientiousness, agreeableness, and emotional stability). A one-point increase in authoritarian measurement scores was associated with a decrease in the standardized conscientiousness score by 0.110 SD (*p* < 0.01), a decrease in the agreeableness score by 0.062 SD (*p* < 0.05), and a decrease in the emotional stability score by 0.053 SD (*p* < 0.01). The correlations between the six specific parenting style dimensions of authoritative and authoritarian are presented in Supplementary Table 2A, and the results are consistent with those in Table 3.

**Table 3:** Association between two parenting styles dimensions and student’s non-cognitive abilities.

	Conscientiousness	Extroversion	Agreeableness	Openness	Emotional stability
Authoritative	0.108**	0.069**	0.101**	0.123**	0.052
	(0.011)	(0.016)	(0.016)	(0.006)	(0.027)
Adj. *R*^2^	0.054	0.021	0.024	0.075	0.052
Authoritarian	−0.110**	0.008	−0.062*	0.002	−0.053**
	(0.006)	(0.012)	(0.022)	(0.011)	(0.007)
Adj. *R*^2^	0.054	0.019	0.021	0.068	0.052
Controls	YES	YES	YES	YES	YES
Class fixed effect	YES	YES	YES	YES	YES
Observations	6,549	6,549	6,549	6,549	6,549

Each cell is a separate regression. Five sub-scales of non-cognitive abilities score are standardized score. Controls included student age (in years), gender, Hukou, minority; family size, number of siblings of child, whether the student’s parent was the primary caregiver, whether the household of the child is poverty-stricken households, educational attainment of father and mother, and a factor of household wealth. Class fixed effects added. All standard errors account for clustering at the class level. *p < 0.05; **p < 0.01.

Table 4 demonstrates that both authoritative and authoritarian parenting styles are significantly associated with specific sub-index scales of non-cognitive development. Specifically, an authoritative parenting style was significantly and positively associated with conscientiousness, extroversion, agreeableness, and openness. A one-point increase in authoritative parenting style was associated with a 0.099 SD (*p* < 0.01) increase in the standardized conscientiousness score, a 0.070 SD (*p* < 0.01) increase in the standardized extroversion score, a 0.096 SD (*p* < 0.01) increase in the standardized agreeableness score, and a 0.124 SD (*p* < 0.01) increase in the standardized openness score. However, authoritarian parenting style was significantly associated with a narrower range of primary index scales than authoritative parenting style, including conscientiousness and emotional stability. Specifically, a one-point increase in authoritarian parenting style was associated with decreases in standardized scores for conscientiousness by 0.100 SD (*p* < 0.01) and emotional stability by 0.048 SD (*p* < 0.01).

**Table 4:** Association between parenting style and student’s non-cognitive ability.

	Conscientiousness	Extroversion	Agreeableness	Openness	Emotional stability
**Parenting style**					
Authoritative	0.099**	0.070**	0.096**	0.124**	0.048
	(0.013)	(0.015)	(0.015)	(0.007)	(0.028)
Authoritarian	−0.100**	0.015	−0.053	0.014	−0.048**
	(0.006)	(0.013)	(0.023)	(0.014)	(0.006)
Controls	YES	YES	YES	YES	YES
Class fixed effect	YES	YES	YES	YES	YES
Adj. R^2^	0.059	0.021	0.026	0.075	0.053
Observations	6,549	6,549	6,549	6,549	6,549

Five sub-scales of non-cognitive abilities score are standardized score. Controls included student age (in years), gender, Hukou, minority; family size, number of siblings of child, whether the student’s parent was the primary caregiver, whether the household of the child is poverty-stricken households, educational attainment of father and mother, and a factor of household wealth. Class fixed effects added. All standard errors account for clustering at the class level. *p < 0.05; **p < 0.01.

#### 3.3.2 Parenting style and students’ non-cognitive outcomes for boys versus girls

The results of the regression analysis on different genders are presented in Table 5, confirming the significant impact of parenting style on non-cognitive development. The extent of the effect varies somewhat between male and female student groups: authoritative parenting style has a significant influence on several outcomes (conscientiousness and extroversion scores) for girls but has little effect on boys. Specifically, the coefficient on the interaction term (−0.045, −0.082) indicates a statistically significant difference in conscientiousness and extroversion scores between boys and girls (*p* < 0.01), suggesting that an authoritative parenting style was associated with conscientiousness and extroversion scores in a significantly different way among boys and girls. The difference in emotional stability scores between boys and girls was statistically significant (*p* < 0.01), as indicated by the coefficient on the interaction term (−0.067), suggesting that an authoritarian parenting style was associated with emotional stability scores in a significantly different way for boys and girls.

**Table 5:** Association between parenting style and students’ non-cognitive ability based on subsamples.

	Conscientiousness	Extroversion	Agreeableness	Openness	Emotional stability
Gender	0.447*	0.495**	0.003	0.626**	0.659**
(1 = male; 0 = female)	(0.119)	(0.054)	(0.154)	(0.104)	(0.094)
Authoritative	0.120**	0.110**	0.104**	0.167**	0.071*
	(0.017)	(0.014)	(0.013)	(0.034)	(0.018)
Authoritative × gender	−0.045**	−0.082**	−0.019	−0.090	−0.048
	(0.010)	(0.016)	(0.063)	(0.066)	(0.028)
Authoritarian	−0.082**	−0.053**	−0.077**	−0.047*	−0.015
	(0.012)	(0.009)	(0.018)	(0.015)	(0.014)
Authoritarian × gender	−0.035	−0.075	0.051	−0.064	−0.067**
	(0.034)	(0.038)	(0.053)	(0.057)	(0.016)
Controls	YES	YES	YES	YES	YES
Class fixed effect	YES	YES	YES	YES	YES
Adj. *R*^2^	0.059	0.022	0.026	0.076	0.054
Observations	6,549	6,549	6,549	6,549	6,549

Five sub-scales of non-cognitive abilities score are standardized score. Controls included student age (in years), Hukou, minority; family size, number of siblings of child, whether the student’s parent was the primary caregiver, whether the household of the child is poverty-stricken households, educational attainment of father and mother, and a factor of household wealth. Class fixed effects added. All standard errors account for clustering at the class level. *p < 0.05; **p < 0.01.

#### 3.3.3 Four parenting style categories and students’ non-cognitive outcomes

The distribution of the four groups of combined parenting styles is presented in Supplementary Table 3A. Among the 6,549 students and their caregivers, 1825 primary caregivers (27.87%) exhibited a mainly authoritative parenting style (Group 1: highly authoritative, lowly authoritarian). Group 2 (high authoritarian, low authoritative) included 2074 families (31.67%), where the primary caregivers mostly adopted an authoritarian approach. Thousand five hundred and eighty-three primary caregivers (24.17%) reported using a style that was both authoritative and authoritarian when raising their children (Group 3: highly authoritative and highly authoritarian). Neither an authoritative nor an authoritarian caregiving style was found in the fourth group, which included 1,067 primary caregivers (16.29%).

The correlations between the four groups of combined parenting styles and students’ non-cognitive development are reported in Table 6. The results presented in Table 6 indicate that students with primary caregivers who belonged to Group 1 had substantially higher scores on several non-cognitive measures than those with caregivers who belonged to Group 4. Specifically, these students had higher scores on the conscientiousness score (*β* = 0.149, *p* < 0.01), extroversion score (*β* = 0.129, *p* < 0.05), agreeableness score (*β* = 0.129, *p* < 0.05), and openness score (*β* = 0.204, *p* < 0.01). In contrast, students whose primary caregivers belonged to Group 2 exhibited significantly lower non-cognitive scores, including a conscientiousness score (*β* = −0.094, *p* < 0.01), an extroversion score (*β* = −0.058, *p* < 0.01), an agreeableness score (*β* = −0.069, *p* < 0.01), and an openness score (*β* = −0.052, *p* < 0.01). Students whose primary caregivers belonged to Group 3 had higher extroversion scores (*β* = 0.136, *p* < 0.01), agreeableness scores (*β* = 0.070, *p* < 0.01), and openness scores (*β* = 0.200, *p* < 0.01) than students whose primary caregivers belonged to Group 4. Correlations between the four categories of combined parental styles and students’ non-cognitive development are displayed in Supplementary Table 4A, with means serving as dividing lines. The results are consistent with Table 6.

**Table 6:** Association between combinations of parenting styles and student’s non-cognitive abilities.

	Conscientiousness	Extroversion	Agreeableness	Openness	Emotional stability
Group 1(high authoritative, low authoritarian)	0.149**	0.129*	0.129*	0.204**	0.060
	(0.035)	(0.032)	(0.042)	(0.020)	(0.064)
Group 2 (low authoritative, high authoritarian)	−0.094**	−0.058**	−0.069**	−0.052**	−0.020
	(0.019)	(0.009)	(0.013)	(0.008)	(0.029)
Group 3 (high authoritative, high authoritarian)	0.011	0.136**	0.070**	0.200**	−0.001
	(0.020)	(0.020)	(0.009)	(0.005)	(0.054)
Controls	YES	YES	YES	YES	YES
Class fixed effect	YES	YES	YES	YES	YES
Adj. *R*^2^	0.057	0.021	0.025	0.075	0.052
Observations	6,549	6,549	6,549	6,549	6,549

Five sub-scales of non-cognitive abilities score are standardized score. Controls included student age (in years), gender, Hukou, minority; family size, number of siblings of child, whether the student’s parent was the primary caregiver, whether the household of the child is poverty-stricken households, educational attainment of father and mother, and a factor of household wealth. Class fixed effects added. All standard errors account for clustering at the class level. *p < 0.05; **p < 0.01.

#### 3.3.4 Student/demographic variables and parenting style

Table 7 presents the correlation between student and household characteristics and authoritative (or authoritarian) parenting styles. When examining student characteristics, we find that the age of the student and Hukou are significantly associated with an authoritative parenting style. Specifically, a one-year increase in the student’s age corresponds to an increase in the authoritative parenting style score by 0.01 points (*p* < 0.01). Primary caregivers are less likely to adopt an authoritative style if the student has a rural Hukou (*β* = −0.114, *p* < 0.01). Additionally, we discovered gender differences in relation to a caregiver’s authoritarian parenting style. It is more likely that the primary caregivers will adopt an authoritarian style if the student is a boy (*β* = 0.132, *p* < 0.01).

**Table 7:** Association between student and household characteristics and parenting style.

	Authoritative parenting style	Authoritarian parenting style
**Student characteristics**		
(1) Student Age (in years)	0.010**	−0.023
	(0.002)	(0.010)
(2) Student is male (1 = yes; 0 = no)	−0.011	0.132**
	(0.012)	(0.024)
(3) Student is boarding	0.055	−0.021
	(0.022)	(0.021)
(3) Student’s Hukou is Rural (1 = yes; 0 = no)	−0.114**	0.063
	(0.006)	(0.031)
(4) Student’s Minority is Han (1 = yes; 0 = no)	−0.020	−0.049
	(0.030)	(0.024)
**Household characteristics**		
(5) Family size (numbers)	0.011	−0.004
	(0.005)	(0.007)
(6) Siblings (numbers)	−0.050**	0.003
	(0.008)	(0.009)
(7) Primary caregiver is parents	0.013	−0.028*
	(0.015)	(0.008)
(8) Paternal education level	0.054*	−0.036**
(1 = upper secondary education or above; 0 = else)	(0.016)	(0.005)
(9) Maternal education level	0.101**	−0.071*
(1 = upper secondary education or above; 0 = else)	(0.008)	(0.024)
(10) Poverty-stricken household (1 = yes; 0 = no)	−0.052	−0.025
	(0.042)	(0.013)
(11) Family asset index	0.021*	−0.011
	(0.006)	(0.015)
Class fixed effect	Yes	Yes
Adj. *R*^2^	0.025	0.021
Observations	6,549	6,549

All standard errors account for clustering at the class level. *p < 0.05; **p < 0.01.

When examining household characteristics, we found that the number of siblings, the education level of both parents, and family asset value were significantly associated with authoritative parenting. Specifically, if the student has more siblings, primary caregivers are less likely to adopt an authoritative parenting style (*β* = −0.050, *p* < 0.01). Additionally, paternal education level (*β* = 0.054, *p* < 0.05), maternal education level (*β* = 0.101, *p* < 0.01), and primary caregivers from financially well-off households (*β* = 0.021, *p* < 0.05) were positively correlated with an authoritative style of parenting. However, paternal education level (*β* = −0.036, *p* < 0.01), maternal education level (*β* = −0.071, *p* < 0.05), and the parent’s status as the primary caregiver (*β* = −0.028, *p* < 0.05) were negatively correlated with authoritarian parenting style.

## 4 Discussion

This study initially explored the relationship between parenting style and the non-cognitive development of high school students. The findings indicate a strong and positive correlation between an authoritative parenting style and the non-cognitive development of high school students. Conversely, an authoritarian parenting style was found to be associated with contrasting effects. Specifically, students raised with an authoritative style exhibited higher levels of conscientiousness, extraversion, agreeableness, and openness, while those raised with an authoritarian style showed lower levels of conscientiousness and emotional stability. The current study’s findings on this association are consistent with previous research conducted in both developed and urban China (Heaven and Ciarrochi, 2008; Deng and Tong, 2020; Zhang et al., 2020; Kugler et al., 2022). These studies have demonstrated that parents adopting an authoritative parenting style have a deeper understanding of their child’s needs and capabilities, proving effective in fostering the child’s non-cognitive development (Febiyanti and Rachmawati, 2021). In contrast, authoritarian parents employ harsh discipline and strict rules to assert their authority over their children, potentially leading to negative emotional states such as fear, frustration, confusion, and anxiety in the child. Children exposed to an authoritarian parenting style, or its characteristics, are more likely to experience adverse non-cognitive developmental outcomes (Hastings et al., 2007; Mensah and Kuranchie, 2013; Zhang and Qin, 2019).

The two-dimensional analysis revealed that primary caregivers scored highly (*M* = 3.32) on the authoritative parenting style but only moderately (*M* = 2.16) on the authoritarian parenting style. These results align with recent studies conducted in urban China (Xia, 2020; Lin et al., 2022). In comparison to two studies conducted in urban China, this study found a significantly lower use of authoritative parenting and a significantly higher use of authoritarian parenting, as evidenced by statistical tests (*t*-tests) comparing the means of authoritative and authoritarian parenting styles.

The current study investigated the relationship between parenting style and the non-cognitive development of high school students, specifically exploring gender differences. The findings indicated that an authoritative parenting style had positive effects on the non-cognitive developmental outcomes of both male and female high school students. Notably, the authoritative parenting style exhibited a more pronounced influence on the conscientiousness and extroversion of girls compared to boys. This result aligns with findings from other studies (Buchanan et al., 2016; Kugler et al., 2022). Moreover, authoritarian parenting was found to have a significant and negative impact on the non-cognitive abilities of both boys and girls, except for the emotional stability scale, suggesting that authoritarian parenting has a stronger effect on boys’ emotional stability than on girls.

Regarding the relationships between parenting style and non-cognitive development in students, assessed through the four-dimensional framework, a significant and positive correlation was identified in a particular combination of parenting styles (referred to as Group 1) compared to a combined parenting style characterized by the absence of both authoritative and authoritarian practices (referred to as Group 4). Adolescents in Group 1, where primary caregivers employed an authoritative parenting style with minimal reliance on authoritarian practices, exhibited superior non-cognitive developmental outcomes. Conversely, Group 2, characterized by a parenting style predominantly authoritarian with infrequent authoritative behaviors, showed adverse correlations with the non-cognitive development of students. Additionally, students in Group 3, where the main caregiver employed a parenting approach combining both high authoritative and high authoritarian styles, demonstrated improved non-cognitive development, except in the case of emotional stability. Although the four-category method of measuring parenting styles has been less explored in studies on the relationship between parenting style and students’ non-cognitive developmental outcomes, these findings align with existing research indicating a positive association between authoritative parenting and children’s non-cognitive development (Deng and Tong, 2020; Zhang et al., 2020). Conversely, authoritarian parenting has been linked to poorer levels of non-cognitive development in students (Kugler et al., 2022). Interestingly, students raised by primary caregivers employing a combination of authoritative and authoritarian parenting styles scored higher in non-cognitive development (Dornbusch et al., 2016).

In our examination of the correlations between parenting styles and students’ characteristics, we observed a tendency for primary caregivers to adopt an authoritative parenting style as the child grows older, while being less inclined to employ an authoritarian approach. This shift might be attributed to parents gradually relinquishing control over their children’s environments as they age and gain more autonomy in decision-making (Rosen et al., 2008; Hotz and Pantano, 2015). Additionally, our findings revealed that the use of an authoritarian style was more prevalent with boys than with girls. This aligns with previous research indicating that girls are often reasoned with, whereas boys are more likely to face physical punishment (Siegal, 1987; Wang et al., 2021). Notably, caregivers tend to employ an authoritarian parenting approach, as opposed to an authoritative one, when students have rural Hukou. This finding is consistent with Pinki and Singh’s (2013) discovery that parents of urban students demonstrate more emotional warmth and understanding, while parents of rural students exhibit higher levels of rejection, punishment, and subject preferences.

In our exploration of the relationships between parenting styles and household characteristics, we identified notable patterns. Specifically, when primary caregivers have a larger number of children in the family, there is a decreased likelihood of employing an authoritative parenting style for each individual child. This finding aligns with a previous study (Lu and Chang, 2013). Furthermore, when parents, as opposed to grandparents, are not the primary caregivers, there is a higher probability of adopting an authoritarian parenting style. This result is consistent with research conducted on preschool-aged children in rural China, revealing that grandmothers, influenced by traditional Chinese culture, tend to exhibit emotional restraint, discourage warmth, and display reluctance in child-rearing (Wang et al., 2022). Moreover, parents with higher levels of education are more inclined to utilize an authoritative approach rather than an authoritarian one in raising their children. These findings align with prior research (Baumrind, 1971; Chen et al., 2000; Khanam and Nghiem, 2016), suggesting that well-educated parents are more likely to recognize the benefits of an authoritative parenting style and apply it in their child-rearing practices. Additionally, a positive correlation was observed between the family asset index and authoritative parenting, indicating that families with higher levels of wealth were more prone to adopting this style of parenting. This result is in accordance with international studies that have demonstrated a correlation between parents of higher socioeconomic status and a greater tendency to employ warm parenting practices (Cobb-Clark et al., 2019).

The study identified two primary limitations that impede the adoption of authoritative parenting styles in rural China: knowledge constraints and economic limitations. Firstly, a lack of knowledge regarding parenting styles and their impact on children may contribute to the observed deficiency in authoritative parenting and an over-reliance on authoritarian parenting (Xu et al., 2005). More educated parents, compared to those with less education, are more likely to value inductive reasoning and democratic methods of control over power assertion (Chen et al., 2000). Secondly, financial difficulties may exacerbate family conflicts, potentially harming the caregiver’s physical and mental health. This, in turn, has the potential to negatively influence the caregiver’s mindset and parenting approach (Liu and Lachman, 2019).

As the world’s largest developing country, exploring the correlation between parenting styles and the non-cognitive development of high school students in rural areas of China holds significant value. Our findings provide evidence that an authoritative parenting style can effectively enhance the non-cognitive abilities of high school students. Conversely, an authoritarian parenting style, characterized by a lack of emotional contact and stringent demands, has been found to impede the non-cognitive development of children in rural areas of China. Therefore, we recommend that policymakers develop parenting education intervention programs to improve the quality of parenting styles in rural China. The results of our study also suggest that certain groups should be specifically targeted for such programs, notably poorer families, families where parents are not present or are not the primary caregivers, and those in which parents have lower education levels.

Studies of parenting style intervention programs in developed and developing settings have led to significant gains in non-cognitive skills among disadvantaged children (Gertler et al., 2014; Attanasio et al., 2020). Parenting training programs can be established with a set curriculum and with guidance provided by trained teachers, and these interventions offer educational support for parents, facilitating the development of positive parenting attitudes and the acquisition of high-quality childcare abilities. Parenting teachers could be nurses or doctors at local township hospitals, or they could be trained paraprofessionals from the local community. By implementing interventions of this nature, rural caregivers can build knowledge and practical skills to help children to develop their full potential.

This study contributes to the literature in three key ways. Firstly, it represents the inaugural and singular investigation into how parenting styles impact the non-cognitive developmental outcomes of high school students in rural China. The research provides significant and novel insights into how parenting styles may shape the non-cognitive development of children in low- and middle-income rural environments. Earlier studies have indicated that adolescents in such contexts typically undergo adverse non-cognitive developmental effects (Zhou, 2022). Second, this study is the first to examine the association between parenting style and non-cognitive developmental outcomes for high school students using a two-dimensions method and a four-categories approach. Third, this is the first study to investigate the different influences of parenting style on the non-cognitive developmental outcomes of high school students by gender, offering a crucial analysis in this emerging field of study.

## 5 Limitations and future directions

We acknowledge that this study has several limitations. First, caregivers’ self-reports of their parenting style may have been subject to recall bias. Obviously, many previous studies also experienced this limitation. Second, the impact of students’ non-cognitive development on caregivers’ parenting styles was not examined. Therefore, the bidirectional relationship between students’ non-cognitive development and parenting style cannot be identified through this study. Third, while there were associations between parenting style and students’ non-cognitive abilities, we were unable to make causal implications.

The following suggestions for further research are provided with regard to the study’s limitations: First, in order to avoid the influence of recall bias, other approaches, such as observing parent–child interactions or conducting in-person interviews with children, should be used to evaluate parenting style. Second, future research should concentrate on gathering information on student non-cognitive development and parenting style for additional waves and try to demonstrate whether there is a reciprocal connection between parenting style and students’ non-cognitive development. Finally, future research could assess the causal chain of the connections by using longitudinal datasets of parenting style, student characteristics, and the non-cognitive development of students.

## Data availability statement

The raw data supporting the conclusions of this article will be made available by the authors, without undue reservation.

## Ethics statement

The studies involving humans were approved by the Ethics Committee of Renmin University of China. The studies were conducted in accordance with the local legislation and institutional requirements. Written informed consent for participation in this study was provided by the participants’ legal guardians/next of kin.

## Author contributions

SW: Formal analysis, Resources, Writing – original draft, Writing – review & editing, Conceptualization, Funding acquisition. LZ: Data curation, Formal analysis, Investigation, Methodology, Resources, Validation, Visualization, Writing – original draft, Writing – review & editing.

## Footnotes

^1^Authoritative parenting style has been primarily associated with higher academic achievement and better cognitive development and lower school drop-out rates (Spera, 2005; Dornbusch et al., 2016; Wang et al., 2022). The rest three parenting styles have been associated with decreased estimates of completing higher education, lower cognitive ability, and lower academic achievement in high school (Majumder, 2016; Kimmes and Heckman, 2017).

^2^The Big Five personality traits, also referred to as the five-factor model (FFM), is widely acknowledged by psychologists as a reliable taxonomy of personality that can be applied to a variety of theoretical frameworks, methodologies, and cultural contexts (McCrae and Costa, 1987; Mount and Barrick, 1998).

^3^Sample size were as follows: *n* = 1,191 in Kugler et al. (2022), *n* = 431 in Loudová and Lašek (2015).

^4^In large-scale comprehensive surveys in China, as of 2018, there has never been a precedent for adding a personality test to a questionnaire. Although there are a small number of questions related to the personality description of the interviewees in the survey, but they are insufficient lack of systematic personality questionnaire data. In 2018, with a national representative the “China Family Panel Studies” of the representative sample was conducted in its latest issue. The same tests as BFI-S were introduced in the investigation measure the tool and adjust its options.
